# Office orthostatic blood pressure measurements and ambulatory blood pressure monitoring in the prediction of autonomic dysfunction

**DOI:** 10.1186/s40885-016-0059-4

**Published:** 2017-03-15

**Authors:** Kawther F. Alquadan, Girish Singhania, Abhilash Koratala, Abutaleb A. Ejaz

**Affiliations:** 1 Division of Nephrology, Hypertension and Renal Transplantation, University of Florida, P.O. Box 100224, Gainesville, FL 32610-0224 USA; 2 Division of Nephrology and Hypertension, University of Utah, Salt Lake City, USA

**Keywords:** Orthostatic hypotension, Ambulatory blood pressure, Autonomic dysfunction

## Abstract

**Background:**

In this retrospective analysis we investigated the predictive performance of orthostatic hypotension (OH) and ambulatory blood pressure monitoring (ABP) to predict autonomic dysfunction.

**Methods:**

Statistical associations among the candidate variables were investigated and comparisons of predictive performances were performed using Receiver Operating Characteristics (ROC) curves.

**Results:**

Ninety-four patients were included for analysis. No significant correlations could be demonstrated between OH and components of ABP (reversal of circadian pattern, postprandial hypotension and heart rate variability), nor between OH and autonomic reflex screen. Reversal of circadian pattern did not demonstrate significant correlation (*r* = 0.12, *p* = 0.237) with autonomic reflex screen, but postprandial hypotension (*r* = 0.39, *p* = 0.003) and heart rate variability (*r* = 0.27, *p* = 0.009) demonstrated significant correlations. Postprandial hypotension was associated with a five-fold (OR 4.83, CI95% 1.6–14.4, *p* = 0.005) increased risk and heart rate variability with a four-fold (OR 3.75, CI95% 1.3–10.6, *p* = 0.013) increased risk for autonomic dysfunction. Per ROC curves, heart rate variability (0.671, CI_95%_ 0.53–0.81, *p* = 0.025) and postprandial hypotension (0.668, CI_95%_ 0.52–0.72, *p* = 0.027) were among the best predictors of autonomic dysfunction in routine clinical practice.

**Conclusion:**

Postprandial hypotension and heart rate variability on ambulatory blood pressure monitoring are among the best predictors of autonomic dysfunction in routine clinical practice.

## Background

Orthostatic hypotension (OH) is present in a heterogeneous group of disease states. Although not synonymous with autonomic dysfunction, OH is often suggested as a marker of autonomic dysfunction [1]. Patients with OH often have abnormal 24-h ambulatory blood pressure monitoring (ABP) profile characterized by reversal of circadian pattern, postprandial hypotension, and non-compensatory heart rate variability [2]. Interestingly, heightened day-night systolic blood pressure gradient [3], increased nocturnal heart rate [4] and postprandial hypotension [5] on ABP have also been associated with dysfunction of the autonomic nervous system. The diagnosis of autonomic dysfunction requires a battery of testing that includes cardiovagal heart rate tests, laboratory indices of adrenergic function and sudomotor tests. The clinical autonomic tests are complex and usually available only in the clinical neurophysiology laboratory in referral centers. The role of the clinicians is to determine requirement for further testing based on history and physical examination, bedside blood pressure measurements and ABP monitoring. However, it is uncertain whether office orthostatic blood pressure measurements and ABP can reliably predict autonomic dysfunction. The aim of the study was to assess the relationship between OH, ABP and autonomic dysfunction.

## Methods

Database of a previously published study of patients who underwent ABP were reviewed (*N* = 1680) [2]. To investigate the relationship between OH, ABP and autonomic dysfunction, we identified ninety-four patients who had simultaneously undergone office OH measurements, ABP and autonomic reflex screen as part of the investigation for suspected autonomic dysfunction based on symptomatology. Of note, ABP and autonomic reflex screen testing were triggered by symptomatology and not merely by the presence of positive office OH. We then determined the presence or absence of office OH based on multiple measurements. OH was defined as a sustained reduction in systolic blood pressure of 20 mmHg or a diastolic blood pressure of 10 mmHg within three minutes of standing when compared with blood pressure from the sitting or supine position [6]. Blood pressure and heart rate were measured with an ABP monitor (model 90207, Spacelabs Medical, Redmond, WA). Measurements were obtained every 15 min while the patient was awake and every 30 min while sleeping. Patients were instructed to record in a journal the time of the following daily events: sleep and wake times, meal times, and any symptoms. Nocturnal blood pressure was defined as the mean blood pressure from the time at which the patient went to bed until the time of awakening, and the daytime blood pressure was defined as the mean blood pressure during the remaining portion of the day. Twenty-four-hours ABP tracings were assessed for the presence or absence of reversal of circadian pattern, heart rate variability and postprandial hypotension. Reversal of circadian pattern was defined as nocturnal blood pressure equal to or more than daytime blood pressure. Heart rate variability was defined as the presence of either of two conditions: (1) lack of increase in heart rate (<10 beats/min) when systolic blood pressure decreased by more than 20 mmHg or (2) lack of increase in heart rate when diastolic blood pressure decreased by more than 10 mmHg [7]. Postprandial hypotension was defined as a decrease in systolic blood pressure of 20 mmHg within 75 min of eating meals [8].

Autonomic function was evaluated using the autonomic reflex screen consisting of an array of tests that noninvasively evaluate the sudomotor, cardiovagal and adrenergic limbs of the autonomic nervous system [9]. The autonomic test battery consisted of beat-to-beat changes in heart rate and blood pressure with Valsalva maneuver and 80° head-up tilt, a thermoregulatory sweating test, and a quantitative sudomotor axon reflex test. The degree of autonomic dysfunction was graded by use of the Composite Autonomic Scoring System (CASS), which summates sudomotor (3 points), cardiovagal (3 points), adrenergic (4 points) deficits on a 10-point scale, with zero meaning no dysfunction. For analysis, individuals were designated as having autonomic dysfunction (CASS score of 1–10) or not (CASS score 0). The CASS has been found to be specific and sensitive for detecting and quantitating symptomatic autonomic failure. A detailed discussion of the clinical autonomic testing is available at American Academy of Neurology website at https://www.aan.com/Guidelines/home/GetGuidelineContent/39.

All results are presented as mean ± standard deviation (or standard error of mean) with *p*-value. *P* value <0.05 was considered statistically significant. Continuous variables were compared with a two-sample t-test or Wilcoxon rank sum test and dichotomous variables with chi-squared test or Fisher’s exact test. Potential, clinically meaningful determinants of autonomic dysfunction were investigated in a univariate screening procedure using Pearson correlation of coefficients (r) test. The nonparametric Spearman rho coefficient of correlation was used to assess correlations between variables without normal distribution. Significant determinants identified from this analysis were studied in a multiple regression model. Area Under the Curve (AUC) of the Receiver-Operating Characteristic (ROC) curves was used to compare the predictive performance of OH, reversal of circadian pattern, postprandial hypotension and heart rate variability to detect autonomic dysfunction. All analyses were conducted using SPSS version 20, Chicago Ill., USA. The study conception, design, execution, data collection, analysis, and manuscript preparation were performed in its entirety and independently by the investigators. All authors had independent access to data and analysis. Institutional Review Board’s requirement of approval was waived for this previously published dataset.

## Results

### Patient characteristics

OH was present per office blood pressure measurements in 77.6% of the patients. Lightheadedness or dizziness was the presenting symptoms in 36.4% of the patients. The characteristics of the patients are shown in Table 1. Patients were predominantly Caucasians, reflecting the local referral pattern, and included more males than females. Comorbid conditions included hypertension, various types of cancers (head and neck 33.3%, genitourinary 47.6%, gastrointestinal 9.5%, other 9.5% of all cancers) and neurological disorders (Parkinson’s disease 71.4% and multi-system atrophy 28.5% of all neurological diagnoses). Mean office supine blood pressure was 152 ± 7/84 ± 5 mmHg and standing blood pressure was 115 ± 2/61 ± 1 mmHg. Mean fall in supine to standing systolic blood pressure (degree of orthostatic fall, deltaOH) was 36.8 ± 0.7 mmHg. Two patients were taking beta-blocker medications and three patients were on alpha-2 adrenergic receptor agonists. None of the patients were on diuretics, renin-angiotensin-aldosterone system blockers, calcium channel blockers or vasodilators.

**Table 1:** Patient characteristics

Demographics	*N* = 94
Age (years)	71 ± 0.9
Male gender (%)	56.4
Caucasian race (%)	98.9
Comorbid conditions	
Hypertension (%)	38.3
Diabetes (%)	9.6
Coronary artery disease (%)	18.1
Hyperlipidemia (%)	12.8
Cancer (%)	22.3
Neurological disorder (%)	14.9
Stroke (%)	8.5
Blood pressures	
Supine (mean ± STD, mmHg)	152 ± 7/84 ± 5
Standing (mean ± STD, mmHg)	115 ± 2/61 ± 1

*STD* = standard deviation

### Orthostatic and ambulatory blood pressure monitoring

The contributions of the individual components of ABP are shown in Table 2. Reversal of circadian pattern and postprandial hypotension were the most common abnormalities among the three components of ABP. Statistically significant correlations between OH and reversal of circadian pattern (*r* = 0.04, *p* = 0.687), postprandial hypotension (*r* = 0.02, *p* = 0.864 or heart rate variability (*r* = 0.03, *p* = 0.792) could not be demonstrated. Neither were any significant correlations between the deltaOH and reversal of circadian pattern (*r* = 0.03, *p* = 0.758), postprandial hypotension (*r* = 0.12, *p* = 0.232) or heart rate variability (*r* = 0.16, *p* = 0.118).

**Table 2:** Contributions of the components of ambulatory blood pressure monitoring and autonomic reflex screen in orthostatic hypotension

Ambulatory blood pressure monitoring	
Reversal of circadian pattern (%)	81.9
Postprandial hypotension (%)	75.5
Heart rate variability (%)	60.6
Autonomic reflex screen	
Sudomotor (%)	86.1
Cardiovagal (%)	80.5
Adrenergic (%)	91.6

### Orthostatic blood pressure and autonomic reflex screen

Autonomic reflex screen, according to the CASS score, was positive in 80.8% (76/94) of the patients. The mean CASS score was 1.9 ± 0.2, median 2.0 (range 9, min 0, max 9). No significant correlation could be demonstrated between OH and autonomic reflex screen (0.13, *p* = 0.217) or CASS score (*r* = 0.05, *p* = 0.646), nor between deltaOH and autonomic reflex screen (*r* = 0.01, *p* = 0.965) or CASS score (*r* = 0.06, *p* = 0.584).

### Ambulatory blood pressure monitoring and autonomic reflex screen

Examination of the components of ABP showed that reversal of circadian pattern did not demonstrate significant correlation with autonomic reflex screen (*r* = 0.12, *p* = 0.237) or to individual components of autonomic reflex screen (sudomotor, *r* = −0.2, *p* = 0.372; cardiovagal, *r* = 0.07, *p* = 0.666; and adrenergic, *r* = 0.02, *p* = 0.921). Postprandial hypotension demonstrated significant correlation with CASS score (*r* = 0.24, *p* = 0.012) and autonomic reflex screen (*r* = 0.39, *p* = 0.003). Heart rate variability also showed significant correlations with CASS score (*r* = 0.29, *p* = 0.002) and autonomic reflex screen (*r* = 0.27, *p* = 0.009). The relationship of the sum of the number of abnormal components of ABP (ABP_Total_ = reversal of circadian pattern + postprandial hypotension + heart rate variability) and CASS score and autonomic reflex screen were also examined. Significant correlation was noted between ABP_Total_ and CASS score (*r* = 0.38, *p* <0.001) and autonomic reflex screen (*r* = 0.31, *p* = 0.002). In unadjusted model, reversal of circadian pattern did not significantly increase the risk for autonomic dysfunction (odds ratio, OR 2.32, CI_95%_ 0.7–7.6, *p* = 0.163). In contrast, postprandial hypotension was associated with a five-fold (OR 4.83, CI_95%_ 1.6–14.4, *p* = 0.005) increased risk for and heart rate variability with a four-fold (OR 3.75, CI_95%_ 1.3–10.6, *p* = 0.013) increased risk for autonomic dysfunction. In the adjusted model that included conditions usually associated with autonomic dysfunction (Parkinson’s disease, multi-system atrophy, diabetes), OH and components of ABP, postprandial hypotension was associated with a four-fold (OR 4.36, CI_95%_ 1.4–13.6, *p* = 0.009). Further analysis revealed that in comparison to patients without postprandial hypotension, patients with postprandial hypotension had significantly higher CASS scores (postprandial hypotension 2.0 ± 0.2 vs. No postprandial hypotension 1.3 ± 0.3, *p* = 0.042) but no significant differences in individual components of the autonomic reflex screen test: sudomotor, postprandial hypotension (*p* = 0.795), cardiovagal (*p* = 0.298) and adrenergic (0.852).

In the subgroup analysis of patients without cancer (*N* = 73), reversal of circadian pattern did not demonstrate significant correlation with autonomic reflex screen (*r* = 0.19, *p* = 0.115) or to individual components of autonomic reflex screen (sudomotor, *r* = −0.04, *p* = 0.847; cardiovagal, *r* = 0.11, *p* = 0.552; and adrenergic, *r* = −0.08, *p* = 0.688). Heart rate variability did not demonstrate significant correlation with autonomic reflex screen (*r* = 0.19, *p* = 0.102) or to individual components of autonomic reflex screen (sudomotor, *r* = 0.19, *p* = 0.288; cardiovagal, *r* = 0.12, *p* = 0.533; and adrenergic, *r* = −0.17, *p* = 0.349). Postprandial hypotension did not demonstrate significant correlation with individual components of autonomic reflex screen (sudomotor, *r* = 0.19, *p* = 0.795; cardiovagal, *r* = 0.16, *p* = 0.386; and adrenergic, *r* = −0.04, *p* = 0.841). Similar to results in the full cohort above, postprandial hypotension and ABP_Total_ demonstrated significant correlation with autonomic reflex screen (*r* = 0.31, *p* = 0.007 and *r* = 0.31, *p* = 0.007, respectively) and CASS score (*r* = 0.37, *p* = 0.001 and *r* = 0.25, *p* = 0.030, respectively). We also analyzed the patients with diabetes (*N* = 9). No significant correlations could be demonstrated between ABP (or components) and autonomic reflex screen (or individual components) or with CASS score.

### Performance of orthostatic and ambulatory blood pressure monitoring to predict autonomic dysfunction

**Fig. 1:**
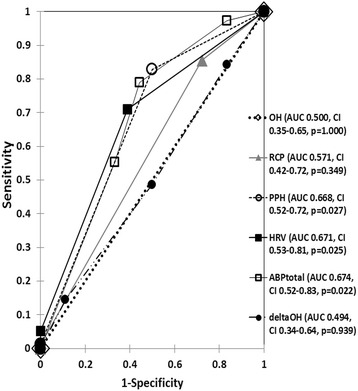
Comparison of ROC curves of orthostatic blood pressure and components of ambulatory blood pressure monitoring to predict autonomic dysfunction. OH: orthostatic hypotension; RCP: reversal of circadian pattern; PPH: postprandial hypotension; HRV: non-compensatory heart rate variability; ABPtotal: sum of the number of abnormal components of ambulatory blood pressure monitoring; deltaOH: degree of orthostatic fall

Comparison of AUCs of OH, deltaOH, ABP_Total_ , reversal of circadian pattern, postprandial hypotension and heart rate variability are shown in Fig. 1. Heart rate variability and postprandial hypotension had the best predictive performance, deltaOH did not demonstrate favorable performance. Although the predictive performance improved when the sum of the abnormal ABP components (ABP_Total_) were utilized, postprandial hypotension and heart rate variability independently demonstrated comparable predictive power. In the subset of patients with neurological disorders (*N* = 14) comparison of AUCs were as follows: OH- 0.500 (0.00–1.00, *p* = 1.000), deltaOH- 0.885 (0.67–1.00, *p* = 0.215), ABPTotal- 0.385 (0.00–0.88, *p* = 0.710), reversal of circadian pattern- 0.500 (0.00–1.00, *p* = 1.000), postprandial hypotension- 0.462 (0.00–1.00, *p* = 0.901) and heart rate variability- 0.500 (0.00–1.00, *p* = 1.000), respectively. In the subset of patients with non-neurological disorders (*N* = 80), ABP_Total_ (AUC 0.684, 0.53–0.84, *p* = 0.021), postprandial hypotension (AUC 0.673, 0.52–0.81, *p* = 0.029) and heart rate variability (AUC 0.665, 0.52–0.81, *p* = 0.037) demonstrated best predictive performances followed by reversal of circadian pattern (AUC 0.568, 0.41–0.73, *p* = 0.394), OH (AUC 0.500, 0.34–0.66, *p* = 1.000) and deltaOH (AUC 0.453, 0.30–0.61, *p* = 0.556), respectively. In the subset that included neurological or diabetes patients (*N* = 21), AUCs of the variables were as follows: OH- 0.500 (0.07–0.93, *p* = 1.000), deltaOH- 0.833 (0.66–1.00, *p* = 0.127), ABP_Total_- 0.333 (0.02–0.65, *p* = 0.445), reversal of circadian pattern- 0.452 (0.08–0.82, *p* = 0.827), postprandial hypotension- 0.452 (0.05–0.85, *p* = 0.202) and heart rate variability- 0.429 (0.08–0.77, *p* = 0.743), respectively.

## Discussion

OH is a common clinical finding, occurring in 5% in patients <50 years of age to 30% in those >70 years of age [10]. OH is associated with frequent falls [11], increased risk for the non-cardiovascular disease mortality [12] and autonomic dysfunction [13, 14]. Although ABP is utilized to detect and assess the severity of OH associated with autonomic dysfunction, its ability to predict underlying autonomic dysfunction is uncertain. In this study, we investigated the predictive performance of office orthostatic blood pressure measurements and ABP monitoring to predict autonomic dysfunction.

The major findings of the study were that two of the ABP components, i.e., heart rate variability and postprandial hypotension were major predictors of and were associated with a four- and five-fold increased risk for autonomic dysfunction, respectively. Interestingly, heart rate variability was the least common among ABP findings, present in only 60% of the patients. Heart rate variability has been well documented in diabetes, hypertension, chronic kidney disease and conditions associated with autonomic dysfunction including in paraplegics [15–17]. Postprandial hypotension has been reported to closely relate to somatic and autonomic neuropathy and was present in 37% of the diabetic patients on ABP [18], in 83% of patients with OH^2^ and in 100% of Parkinson’s disease patients [19]. Perhaps the role of the autonomic nervous system in postprandial hypotension is better demonstrated by the finding that in order to prevent it, a more than 200% increase in sympathetic nervous activity during eating is required compared with that of the mean daytime activity, thus making eating a significant cardiovascular load in those with autonomic dysfunction [20]. Postprandial hypotension is prevalent in the elderly, geriatric population but is not commonly diagnosed [21] perhaps due to the tendency to focus more on reversal of circadian pattern and heart rate variability when interpreting ABP reports.

An important finding was the lack of correlation of reversal of circadian pattern with autonomic dysfunction, considering that reversal of circadian pattern and autonomic dysfunction are primary hallmarks of many diseases with abnormal neurohormonal regulation [22, 23] and has been linked to end-organ damage [2], insulin resistance [24], increased cardiovascular events and mortality [25]. Secondary analysis of data from the X-CELLENT study has also associated autonomic dysfunction with a heightened day-night systolic blood pressure gradient and more variable systolic blood pressure over 24 h in patients with essential hypertension [3]. In the current study we were unable to control for dietary salt intake, smoking, obesity and obstructive sleep apnea [26], factors that are known to influence reversal of circadian pattern. Another intriguing possibility for our observation maybe the suggestion from ad hoc analysis of the PROOF study data that suggests that autonomic dysfunction precedes an insufficient decrease in nocturnal blood pressure independent of hypertension status, i.e., the time interval [27].

We could not demonstrate significant correlation of OH or deltaOH with ABP or autonomic dysfunction. Others have reported that approximately 83% of patients with OH showed at least one generalized autonomic failure of sympathetic adrenergic and parasympathetic cardiovagal functions [13]. This discrepancy illustrates that OH, although a dramatic presentation, is not synonymous with autonomic dysfunction and represents heterogeneous disease states. In one report, 93% of patients with OH had autonomic dysfunction and their etiology included neurologic diseases in 38% of the cases, diabetes 11%, and the rest were cardiovascular, neoplasm, carotid artery disease, paraproteinemia and obstructive sleep apnea [2].

Bedside observations in clinical medicine trigger investigations to uncover etiology of the patients’ signs and symptoms and can lead to further clinical and basic science research to understand their mechanisms. However, associations are not causative and findings in a particular illness often cannot be extrapolated to a different disease state. Such is the case for orthostatic hypotension that is often used synonymously to denote autonomic dysfunction and to infer the presence of reversal of circadian pattern. In this study the majority of the diagnosis of OH was established in the primary care settings and there was no correlation of orthostatic hypotension with ambulatory blood pressure monitoring and with autonomic dysfunction. It was not the reversal of circadian pattern, but the often overlooked postprandial hypotension and heart rate variability that were strong predictors of autonomic dysfunction.

A limitation of this study was the underrepresentation of hypertension patients who underwent autonomic reflex screen testing and the severity of OH. A simple explanation is that neurologically asymptomatic, hypertension patients are not usually referred for autonomic reflex screen testing. However, we have previously reported that in age-matched hypertension patients reversal of circadian pattern, postprandial hypotension and heart rate variability were present in 15, 2 and 1%, respectively compared to 80, 80 and 75% respectively in patients with OH.^2^ Another issue concerns the accuracy of heart rate variability derived from photoplethysmographic versus electrocardiographic signals. Although comprehensive investigations of all heart rate variability indices in large populations have yet to be performed, recent report suggests sufficient accuracy between them [28]. The analysis of specific subsets strengthens the credibility of our findings but the validation is restricted by the small sample. We were unable to investigate the relationship between variants of OH (initial OH, delayed OH). Despite the limitations of a retrospective analysis, we were able to compare head-to-head office orthostatic blood pressure measurements with ABP and sophisticated methods of testing of the autonomic nervous system.

## Conclusion

We have demonstrated that postprandial hypotension and heart rate variability on ambulatory blood pressure monitoring are strong predictors of autonomic dysfunction in routine clinical practice. Further studies are warranted.

## References

[1] Metzler M, Duerr S, Granata R, Krismer F, Robertson D, Wenning GK (2013). Neurogenic orthostatic hypotension: pathophysiology, evaluation, and management. J Neurol.

[2] Ejaz AA, Haley WE, Wasiluk A, Meschia JF, Fitzpatrick PM (2004). Characteristics of 100 consecutive patients presenting with orthostatic hypotension. Mayo Clin Proc.

[3] Zhang Y, Agnoletti D, Blacher J, Safar ME (2012). Blood pressure variability in relation to autonomic nervous system dysregulation: the X-CELLENT study. Hypertens Res.

[4] Pilleri M, Levedianos G, Weis L, Gasparoli E, Facchini S, Biundo R (2014). Heart rate circadian profile in the differential diagnosis between Parkinson disease and multiple system atrophy. Parkinsonism Relat Disord.

[5] Loew F, Gauthey L, Koerffy A, Herrmann FR, Estade M, Michel JP (1995). Postprandial hypotension and orthostatic blood pressure responses in elderly Parkinson’s disease patients. J Hypertens.

[6] Freeman R, Wieling W, Axelrod FB, Benditt DG, Benarroch E, Biaggioni I (2011). Consensus statement on the definition of orthostatic hypotension, neurally mediated syncope and the postural tachycardia syndrome. Clin Auton Res.

[7] Streeten DHP, Izzo JI, Black HR (1999). Management of orthostatic hypotension, hypertension, and tachycardia. Hypertension Primer: The essentials of high blood pressure.

[8] O’Mara G, Lyons D (2002). Postprandial hypotension. Clin Geriatr Med.

[9] Low PA (1993). Composite autonomic scoring scale for laboratory quantification of generalized autonomic failure. Mayo Clin Proc.

[10] Ricci F, De Caterina R, Fedorowski A (2015). Orthostatic Hypotension: Epidemiology, Prognosis, and Treatment. J Am Coll Cardiol.

[11] Rascol O, Perez-Lloret S, Damier P, Delval A, Derkinderen P, Destée A, et al. Falls in ambulatory non-demented patients with Parkinson’s disease. J Neural Transm. 2015; Apr 7. [Epub ahead of print]. 10.1007/s00702-015-1396-2 25845678

[12] Veronese N, De Rui M, Bolzetta F, Zambon S, Corti MC, Baggio G, et al. Orthostatic Changes in Blood Pressure and Mortality in the Elderly: The Pro.V.A Study. Am J Hypertens. 2015; Mar 11. [Epub ahead of print]. 10.1093/ajh/hpv022 25767137

[13] Kim HA, Yi HA, Lee H (2014). Spectrum of autonomic dysfunction in orthostatic dizziness. Clin Neurophysiol.

[14] Wüllner U, Schmitz-Hübsch T, Antony G, Fimmers R, Spottke A, Oertel WH, Deuschl G (2007). Autonomic dysfunction in 3414 Parkinson’s disease patients enrolled in the German Network on Parkinson’s disease (KNP e.V.): the effect of ageing. Eur J Neurol.

[15] Istenes I, Körei AE, Putz Z, Németh N, Martos T, Keresztes K (2014). Heart rate variability is severely impaired among type 2 diabetic patients with hypertension. Diabetes Metab Res Rev.

[16] Barletta GM, Flynn J, Mitsnefes M, Samuels J, Friedman LA, Ng D (2014). Heart rate and blood pressure variability in children with chronic kidney disease: a report from the CKiD study. Pediatr Nephrol.

[17] Rosado-Rivera D, Radulovic M, Handrakis JP, Cirnigliaro CM, Jensen AM, Kirshblum S (2011). Comparison of 24-h cardiovascular and autonomic function in paraplegia, tetraplegia, and control groups: implications for cardiovascular risk. J Spinal Cord Med.

[18] Sasaki E, Kitaoka H, Ohsawa N (1992). Postprandial hypotension in patients with non-insulin-dependent diabetes mellitus. Diabetes Res Clin Pract.

[19] Ejaz AA, Sekhon IS, Munjal S (2006). Characteristic findings on 24-h ambulatory blood pressure monitoring in a series of patients with Parkinson’s disease. Eur J Intern Med.

[20] Masuda Y, Kawamura A (2003). Role of the autonomic nervous system in postprandial hypotension in elderly persons. J Cardiovasc Pharmacol.

[21] Vloet LC, Pel-Little RE, Jansen PA, Jansen RW (2005). High prevalence of postprandial and orthostatic hypotension among geriatric patients admitted to Dutch hospitals. J Gerontol A Biol Sci Med Sci.

[22] Azuma T, Uemichi T, Funauchi M, Nagai Y, Matsubara T (2002). Ambulatory blood pressure monitoring in patients with spinocerebellar degeneration. Acta Neurol Scand.

[23] Kitae S, Murata Y, Tachiki N, Okazaki M, Harada T, Nakamura S (2001). Assessment of cardiovascular autonomic dysfunction in multiple system atrophy. Clin Auton Res.

[24] Chen JW, Jen SL, Lee WL, Hsu NW, Lin SJ, Ting CT (1998). Differential glucose tolerance in dipper and nondipper essential hypertension: the implications of circadian blood pressure regulation on glucose tolerance in hypertension. Diabetes Care.

[25] Kario K, Motai K, Mitsuhashi T, Suzuki T, Nakagawa Y, Ikeda U (1997). Autonomic nervous system dysfunction in elderly hypertensive patients with abnormal diurnal blood pressure variation: relation to silent cerebrovascular disease. Hypertension.

[26] Rodríguez-Colón S, He F, Bixler EO, Fernandez-Mendoza J, Vgontzas AN, Berg A (2014). The circadian pattern of cardiac autonomic modulation and obesity in adolescents. Clin Auton Res.

[27] Dauphinot V, Gosse P, Kossovsky MP, Schott AM, Rouch I, Pichot V (2010). Autonomic nervous system activity is independently associated with the risk of shift in the non-dipper blood pressure pattern. Hypertens Res.

[28] Schäfer A, Vagedes J (2013). How accurate is pulse rate variability as an estimate of heart rate variability? A review on studies comparing photoplethysmographic technology with an electrocardiogram. Int J Cardiol.

